# Editorial: Bioactive Compounds Biosynthesis and Metabolism in Fruit and Vegetables

**DOI:** 10.3389/fpls.2020.00129

**Published:** 2020-02-19

**Authors:** Alessandra Francini, Manuela Pintado, George A. Manganaris, Antonio Ferrante

**Affiliations:** ^1^ Institute of Life Sciences, Scuola Superiore Sant'Anna, Pisa, Italy; ^2^ Escola Superior de Biotecnologia, Universidade Católica Portuguesa, Porto, Portugal; ^3^ Department of Agricultural Sciences, Biotechnology & Food Science, Cyprus University of Technology, Lemesos, Cyprus; ^4^ Department of Agricultural and Environmental Sciences—Production, Landscape, Agroenergy, Università degli Studi di Milano, Milan, Italy

**Keywords:** carotenoids, phenolics, abiotic stress, vitamin, anthocyanin, breeding, hormonal regulation

## Bioactive Compounds Biosynthesis and Metabolism in Fruit and Vegetables

Fruit and vegetables are considered to be among the most important sources of bioactive compounds with proven beneficial effect on human diet. Tomato has been evolved as a model crop to study both fruit ripening pattern as well as for understanding how different environmental and agricultural factors can enhance the accumulation of bioactive compounds. The concentration of bioactive compounds is highly dependent on the crop species, cultivar/genotype, agronomic management, preharvest environmental conditions, and postharvest management practices (Toscano et al.). Bioactive compounds in fruit and vegetables are of consumer interest for their potential benefit to the health, especially in counteracting several diseases related to aging and stress. However, the bioactive molecules also have preservation properties that extend the shelf life of the produce. Postharvest technologies and storage conditions can reduce the degradation of bioactive compounds and some industrial operation can even promote their accumulation.

The systematic screening of key bioactive compounds with high content in a wide range of germplasm and the restoration of key genes and gene clusters from wild species and/or landraces both are important for reducing the loss of agro-biodiversity and the creation of a “gene pool” that can be exploited in future breeding programs toward the release of new cultivars with added nutraceutical value (Manganaris et al., 2018). Furthermore, the understanding how the accumulation of bioactive compounds can be enhanced or preserved is crucial for improvement of crop and product quality (Toscano et al.). The availability of advanced molecular tools allows fast and accurate transcriptome profiling that can help in the identification of the main gene clusters that are activated or repressed under different conditions. Such information coupled with the big data from metabolomics studies will be useful both for preharvest and postharvest management of produce with high nutritional value.

## Impact of Genotype and Significance of ‘Old-Fashioned’ Cultivars on Bioactive Content

Bioactive compounds show wide variations among different species. Several studies have reported that ancient or old varieties accumulate higher concentration of bioactive compounds that have been linked to their adaptation to different environmental conditions (García-Mier et al., 2013; Manganaris et al., 2018). The accumulation of bioactive compounds can serve as defenses against biotic and abiotic stresses. Local varieties of tomato (*Solanum lycopersicum* L.) grown in Tuscany showed higher polyphenols, flavonoids, and carotenoids compared with commercial ones (Berni et al.). Similar results have been reported in onion (*Allium cepa* L.) and sweet cherry [*Prunus avium* L., (Berni et al.)]. In the meantime, breeding projects are targeting toward the release of new cultivars with enhanced bioactive content, as this is the case for tomatoes (Lenucci et al., 2006). Recently, interest for the black tomatoes has been attributed to the high carotenoid and anthocyanin contents. The ‘Sun Black’ tomato, a result of a 20-years breeding activity, derived from wild tomato species, such as *Solanum chilense, Solanum cheesmaniae*, *Solanum lycopersicoides,* and *Solanum habrochaites,* through introgression with cultivated genotypes (Mazzucato et al., 2013). A comparative study showed that the anthocyanins-enriched ‘Sun Black’ tomato had an almost double concentration of phenolics and carotenoids at the ripe stage compared to the wild type. Color is often an indicator of the composition and concentration of the bioactive compounds. In tomatoes and watermelon, the red color is due to the accumulation of lycopene while the yellow color due to β-carotene. The concentration of these two carotenoids can induce different flesh and skin colors (Ilahy et al.). These compounds are also substrates for volatile biosynthesis, contributing to fruit aroma with direct effect on produce quality (Ilahy et al.).

## Environmental Conditions Affect the Accumulation of Bioactive Compounds

Environmental conditions can positively or negatively affect the concentration of bioactive molecules in different horticultural produce. Tomato fruits obtained from plants exposed to high salinity conditions (60 or 120 mM NaCl) showed a reduction of antioxidant capacity and several secondary metabolites such as lycopene and phenols (Moles et al.).

Environmental conditions including altitude, temperature, and light can influence bioactive compound accumulation. A study carried out on blueberry (*Vaccinium corymbosum* L.) grown in different altitudes, was shown that lower altitudes induced an early ripening and a higher anthocyanin accumulation (Spinardi et al.).

Light quality can induce the biosynthesis of different secondary metabolites that can have protective functions against biotic and abiotic stresses. Plants exposed to UV-B treatments have increased phenolic compounds in a dose-response manner. In a study performed in peaches (*Prunus persica* L.), UV-B treatments applied for 1 or 3 h enhanced several phenolic compounds. The employment of UV-B was also studied as priming for preventing the development of *Monilinia fructicola* fungus (Santin et al.). Treatments applied for different durations indicated that 1 and 3 h of UV-B treatments increased the phenolics in the fruit except near the inoculation point, while around the inoculation point the effect of UV-B treatments were not always consistent depending also on the effects of fungus, the wounding and their interaction (Santin et al.).

## Hormonal Regulation of Bioactive Compounds

There are plant hormones that have bioactive molecules as precursors such as abscisic acid (ABA), auxin, salicylic acid (SA), and melatonin. ABA biosynthesis is derived from the catabolism of carotenoids, while the auxins, SA, and melatonin are synthetized from the chorismite as their precursor. The connecting molecules may explain the cross-talks among them and their roles in the modulation of the growth and the ripening process of both climacteric and non-climacteric fruits (Pérez-Llorca et al.).

The protection of plant cells from external environments and biotic or abiotic stresses is partly provided by the cell wall and, in several species, by the cuticular waxes. The major components of cuticular wax are very long chain fatty acids and derived compounds (Trivedi et al.). However, the concentration and composition of these molecules in the cuticular waxes varies among species and within cultivars of the same species. Tomato mutants such as NON-RIPENING (*nor*) and RIPENING INHIBITOR (*rin*) have different cuticular waxes (Kosma et al., 2010). These findings suggest that ethylene plays a role in the wax biosynthesis and accumulation. This hypothesis has been confirmed in the apple and orange. At molecular level, it has been shown that many transcription factors are involved in the regulation of wax biosynthesis such as FRUITFULL and TOMATO AGAMOUS-LIKE1 (Trivedi et al.). In particular, the MADS-RIN transcription factor TDR4/FUL1 and its homolog MBP7/FUL2 have high similarity with FRUITFULL of Arabidopsis. This TDR4 transcription factor has been reported to be involved in pigment biosynthesis in tomato fruit. A functional analysis of this gene using virus-induced gene silencing technology (VIGS) demonstrated that the TDR4 gene is effectively involved in the biosynthesis of bioactive molecules. In fact, TDR4-silenced tomatoes showed a strong reduction of amino acids and α-tomatine (Zhao et al.).

The collection of articles in this Research Topic demonstrates that the accumulation of bioactive compounds in produce can derive from different environmental, genetic, and agronomic factors.

## Author Contributions

All authors planned the structure of the editorial, contributed in its writing, read and approved the submitted version.

## Conflict of Interest

The authors declare that the research was conducted in the absence of any commercial or financial relationships that could be construed as a potential conflict of interest.
